# The bottlenecks, architectures and future pathways of optoelectronic integration: an interview with Qionghai Dai

**DOI:** 10.1093/nsr/nwag431

**Published:** 2026-07-11

**Authors:** Jiamin Wu, Jingxuan Chen

**Affiliations:** Department of Automation, Tsinghua University; Institute of Semiconductors, Chinese Academy of Sciences

## Abstract

It has been more than 50 years since the concept of optoelectronic integration was first proposed. The concept refers to the integration of optical and electronic devices on a single chip to realize the combined functionalities of light manipulation and electronic data processing; it is widely considered a key approach to overcoming the speed, power, and bandwidth bottlenecks of conventional electronic systems. Despite the continuous emergence of new materials and applications, large-scale deployment has yet to be realized.

Driven by emerging demands in AI computing, optical computing, and quantum computing, the field now faces a series of fundamental choices: Should we pursue extreme integration scale? Should we target monolithic integration? Should we push toward nanoscale dimensions? NSR spoke with Professor Qionghai Dai from Tsinghua University, an academician of the Chinese Academy of Engineering, to explore core questions regarding the developments, current architectures and future pathways of optoelectronic integration.

## DEVELOPMENTS AND SCALING LIMITS OF OPTOELECTRONIC INTEGRATION

***NSR*:** The concept of optoelectronic integration was first proposed by Yariv *et al.* in 1972. Since then, new materials and concepts have continued to emerge, making it a highly active research field with rich potential applications. However, after more than 50 years of development, we have yet to see widespread commercial deployment and consumer products of optoelectronic integration. What is your perspective on its development trajectory and future prospects?

***Dai*:** The long gap between Yariv’s proposal and broad deployment is not a sign of failure; it reflects that optoelectronic integration is fundamentally a system co-design problem rather than a single device technology. Electronics rode a clean scaling law because charge is easy to confine, switch, and store at the nanoscale. Light is not. It resists confinement below its wavelength, has no efficient native source on silicon, and is hard to store. So progress has been quieter and more application-specific than electronics.

Yet integration has already succeeded where the value is highest: in data communication (datacom) and telecommunication (telecom) transceivers, in coherent optics, and increasingly in co-packaged optics for data centers. The reason we see no consumer handset product is simply that the advantages of integration appear at extreme bandwidth density and energy scales, not yet in pocket-sized devices.

My view is cautiously optimistic. The field is moving from discrete assembly toward wafer-scale and chiplet-level co-integration, and the demand from artificial intelligence is now strong enough to pull the technology from niche to necessity. The risk I would flag is that the community sometimes pursues integration as a goal in itself. The decisive question for the next decade is not how tightly we can integrate, but which applications make integration unavoidable. Those applications, computing and interconnect for large-scale intelligence, are finally arriving.

**Figure 1:**
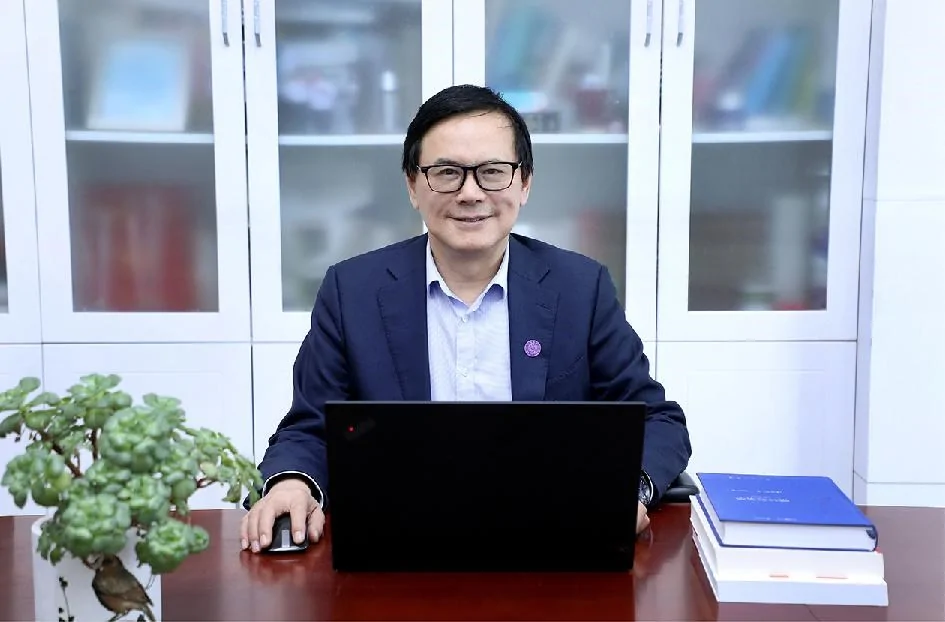
Prof. Qionghai Dai’s work lies at the forefront of optoelectronic integration, spanning from intelligent perception to analog optoelectronic computing. *Photo provided by Prof. Dai.*

***NSR*:** Could you share your views on the scale of optoelectronic integration: What are the key factors limiting its scaling? Is a fundamental breakthrough feasible in the near future? And is it necessary to persistently pursue very-large-scale optoelectronic integration in the long term?

***Dai*:** The hard floor on photonic density is set by the wavelength. A photonic component is typically a few microns, while a transistor is a few nanometers, leaving a footprint gap of two to three orders of magnitude. Layered on top are practical limits: the absence of an efficient on-chip light source on silicon, modulator and detector energy efficiency, thermal sensitivity, optical crosstalk, and the stubborn cost of fiber coupling and packaging.

I do not expect a single dramatic breakthrough that closes this gap. Progress will come incrementally and on several fronts at once: heterogeneous integration that imports III–V gain onto silicon, two-dimensional and phase-change materials, plasmonic and slot confinement for active devices, and advanced packaging that reduces the energy of every electrical-optical crossing.

On the deeper question of whether we should chase very-large-scale integration in the way electronics did, my answer is no, not as a default. Copying the transistor-count roadmap is the wrong instinct. The right figure of merit for photonics is functional density and system value, measured in bandwidth density and energy per bit or per operation, not in component count. For interconnect and computing, a modest number of high-quality optical channels often beats a vast number of marginal ones. We should pursue scale precisely where it serves function, and resist scaling as a slogan.

*The decisive question for the next decade is not how tightly we can integrate, but which applications make integration unavoidable.*

*–—Qionghai Dai*

## INTEGRATION ARCHITECTURES AND SYSTEM-LEVEL CONSIDERATIONS

***NSR*:** Integrated electronic devices have already entered the nanoscale. Do you believe photonic devices also need to advance toward nanoscale dimensions? If so, how can the limitations imposed by their wavelengths be overcome?

***Dai*:** Not uniformly, and I think the framing deserves care. The diffraction limit, roughly half a wavelength in the medium, sets a real floor for conventional dielectric photonics. To go below it one can use plasmonics, high-index and slot waveguides, two-dimensional materials, epsilon-near-zero media, and metasurfaces. But every one of these comes with a cost, usually optical loss, heat, or fabrication difficulty, so shrinking is not free the way it largely was for transistors.

The more useful question is what we actually want from miniaturization: raw density, or improved function. For passive routing, there is little reason to fight the wavelength; micron-scale waveguides are fine. The payoff from going small is concentrated in the active devices, modulators and

*Heterogeneous integration will reach large-scale, high-value application first, and it is already doing so.*

*–—Qionghai Dai*

detectors, where reducing the mode area lowers capacitance and therefore energy per bit and bandwidth. That is where nanoscale or sub-wavelength confinement genuinely earns its place, and where hybrid plasmonic-photonic structures are most justified.

The way to live with the wavelength limit, rather than defeat it everywhere, is to abandon the assumption that photonics and electronics must share the same pitch. Heterogeneous integration lets a few-micron photonic layer sit directly above a nanoscale electronic layer, each at its natural scale, connected vertically. We should miniaturize photonics selectively, where energy and bandwidth demand it, and not force the whole optical layer to match transistor dimensions.

***NSR*:** In your opinion, which integration architecture—monolithic integration, heterogeneous integration, or other types—will be the first to achieve large-scale, high-value practical applications? Is monolithic integration an essential goal for optoelectronic integration?

***Dai*:** I believe heterogeneous integration will reach large-scale, high-value application first, and it is already doing so. The reason is physical, not merely industrial. Silicon has an indirect bandgap and makes a poor light source, so monolithic silicon photonics has always struggled with gain. Heterogeneous integration sidesteps this by letting each material do what it is best at: III–V for gain, silicon for low-loss routing and high-speed modulation, and mature CMOS (Complementary Metal Oxide emiconductor) for control and memory. Co-packaged optics and chiplet-based assembly are the commercial embodiments of this idea today.

Monolithic integration is elegant and, for certain very-high-volume and latency-critical functions, it will remain attractive because it removes interfaces and assembly cost. But I do not regard it as an essential end goal. It is one tool among several. Treating monolithic integration as the destination risks forcing incompatible materials into a single process for reasons of aesthetics rather than performance.

Our own experience with distributed, modular optical computing reinforces this. Scaling a large photonic computing system has been most tractable when we partition the problem across reconfigurable modules rather than insisting on a single monolithic die. The pragmatic path is heterogeneous and modular now, with monolithic reserved for the specific cases where interface elimination genuinely dominates the cost and performance balance.

***NSR*:** Although optoelectronic integration maximizes the complementary functional advantages of optical and electronic chips, it inevitably requires extensive optoelectronic and digital-to-analog conversions, which introduce additional system power consumption and latency. How should the trade-offs between the benefits and costs of integration be balanced? What do you think is the optimal integration architecture: should optical and electronic chips be deeply and seamlessly integrated, or should they function mostly independently and only communicate when necessary?

***Dai*:** The conversion overhead is the central tax of any hybrid optoelectronic system, and the design philosophy should be built around it. The guiding principle is simple to state and hard to execute: minimize the number of domain crossings, and locate each unavoidable crossing where it is cheapest. We should do as much work as possible within one domain before converting, never converting per operation when we can convert per block.

Applied to computing, this means keeping the linear, parallel, high-throughput operations in optics, keeping nonlinearity, control, decision logic, and memory in electronics, and converting only at the coarse boundaries of large compute stages rather than at every step. The most damaging design mistake is to insert a data converter into the inner loop, where its energy and latency overwhelm any optical gain. In our own analog optoelectronic computing work, the key was precisely to combine the analog optical and analog electronic stages so that the digitization bottleneck is deferred and amortized.

So my answer to the framing question is a layered one. Physically, the chips should be deeply and seamlessly integrated through co-packaging, because that reduces parasitics and the energy of each conversion. Logically, however, the domains should remain functionally separate, each doing what it does best and communicating only at coarse granularity. Neither extreme is right: per-operation seamless conversion is too costly, and fully independent operation forfeits the synergy that motivates integration in the first place.

## EMERGING APPLICATIONS AND KEY CHALLENGES

***NSR*:** For emerging applications like optical neuromorphic computation, could you share your insights on its application prospects? How long do you predict it will take for the technology to reach widespread adoption? What might be the main bottlenecks along this journey -are they rooted in physical fundamentals or engineering challenges?

***Dai*:** This is a direction central to my own work, and I am enthusiastic about it but insist on discipline regarding what counts as progress. Optics is exceptional at linear operations, matrix multiplication and convolution realized through diffraction and interference, performed at the speed of light with almost no energy spent on propagation. To my mind, the decisive question for this field is no longer whether a novel optical framework can be demonstrated, but whether such systems deliver real superiority on complete, end-to-end tasks under realistic conditions of noise, power, and latency. The center of gravity should shift from architectural novelty toward practical application.

Our ACCEL (All-analog Chip Combining Electronics and Light) work was built around exactly this conviction. By combining photons and electrons in an all-analog manner, it uses diffractive optical computing as the front-end encoder and feeds the resulting photocurrents directly into an analog electronic stage, removing the analog-to-digital conversion that otherwise dominates the energy and latency budget. This yielded a systemic energy efficiency of around 75 peta-operations per second per watt and a per-frame latency of tens of nanoseconds, more than three orders of magnitude higher in efficiency and more than an order of magnitude faster than state-of-the-art digital processors on the same vision tasks. What matters is not the elegance of the architecture but its practicality for edge scenarios, such as wearable sensing, autonomous perception, and industrial inspection, where power and latency are the binding constraints.

On timeline, I expect domain-specific edge deployment within 5–10 years, with general-purpose optical computing far more distant. The deepest obstacles are physical, namely optical nonlinearity and the absence of optical memory, while reconfigurability and fabrication uniformity are engineering ones. Above all, the community needs a shared quantitative benchmark that compares optoelectronic and digital computing faithfully on full tasks, accounting for every conversion and every overhead, rather than reporting peak optical metrics in isolation.

***NSR*:** With the rapid advancement of artificial intelligence, there is growing interest in using optical interconnects to address the interconnection challenges of computing clusters at the scale of tens of thousands or even hundreds of thousands of GPUs. Do you think this technological path is sustainable? What challenges might it encounter?

***Dai*:** I think this path is not only sustainable but essentially unavoidable. Electrical interconnect hits a wall where reach, bandwidth, and energy cannot be satisfied simultaneously. Copper simply cannot deliver the required bandwidth density over the distances that these clusters span, and the energy per bit becomes prohibitive. Optical interconnect, progressing from co-packaged optics to true optical input-output and eventually optical switching, is the only route that scales acceptably in energy per bit and reach.

The challenges are real and largely at the system and ecosystem level rather than in basic physics. Cost per bit must keep falling steeply. Reliability is the quiet crisis: when a cluster contains hundreds of thousands of optical links, laser failure rates that look negligible per device become a fleet-level problem, which is why approaches that move or share laser sources are attractive. Thermal stability of wavelength-division multiplexing, co-packaging yield, and the maturity of optical circuit switching all need to advance together. There is also an open architectural question of where to stop using electrical links at the shortest reach and hand off to optics.

*This path [using optical interconnects to address the interconnection challenges of artificial intelligence] is not only sustainable but essentially unavoidable.*

*–—Qionghai Dai*

The decisive factor will be whether linear-drive and co-packaged optics can meet the cost and power targets at volume, and whether the industry can agree on standards. I am confident in the trajectory, but it requires coordinated progress across the whole stack, not a single hero device.

***NSR*:** Optical storage issues pose constraints and challenges for many optoelectronic applications. How critical do you consider optical storage to be? Is optoelectronic integration the best route to resolve this problem in the future?

***Dai*:** Optical memory is one of the genuine missing pieces, and I would rank it among the most consequential unsolved problems. Today, both optical computing and optical interconnect repeatedly convert back to electronic memory to hold state, and those conversions dominate latency and energy. For optical computing in particular, the absence of working optical memory forces optical-electrical-optical round trips that erase much of the advantage optics provides in the first place.

I would separate two distinct problems, because conflating them causes confusion. One is long-term, high-density archival storage of data, where optical media have their own trajectory. The other is fast working memory and buffering for optical compute and interconnect, which is the truly limiting issue for the topics in this Special Issue. For the latter, integrated approaches based on phase-change materials and on-chip optical state hold promise but remain immature in density, endurance, and energy.

Is optoelectronic integration the best route to resolve this? For working memory, I expect the answer to be a hybrid rather than an all-optical solution. Optics will handle transport and computation, while state is held using a combination of phase-change elements and electronic memory placed in close co-packaged proximity to minimize conversion cost. All-optical memory at useful scale is not yet proven, so the pragmatic and most likely successful answer is tight integration of complementary technologies, not insistence on a purely optical store.

***NSR*:** To meet the ever-increasing capacity demands of optical interconnection, researchers are dedicated to enhancing single-wavelength bit rates and have achieved remarkable results. However, such ultra-high speeds also impose significant challenges on both optical devices and their supporting electronic components. What is your perspective on the necessity of continuously increasing single-wavelength bit rates and the associated bottlenecks? From both technical and cost perspectives, is there a practical limit or an optimal value?

***Dai*:** There is a strong and legitimate incentive to raise the per-lane rate, because for a given aggregate capacity it reduces the number of lasers, modulators, detectors, and fibers, which in turn lowers cost and footprint. So, the pursuit is rational, not vanity. The difficulty is that the cost of speed does not fall on the optics alone.

As the symbol rate climbs, the burden shifts heavily onto the electronics. Modulator and detector bandwidth become limiting, higher-order modulation demands more signal-to-noise margin, and above all the power of the supporting digital signal processing and the data converters grows faster than linearly with baud rate. Beyond a point, the electronic domain, not the optical channel, sets the energy and cost per bit.

For this reason, I do not believe extreme single-lane rates are the right long-term answer. There is a system-level optimum where the marginal cost of pushing the per-lane rate higher equals the marginal cost of adding parallelism instead, through more wavelengths in wavelength-division multiplexing or more spatial channels. My expectation is that the field will settle on moderate, well-engineered per-lane rates combined with massive parallelism enabled by dense multiplexing and co-packaging, rather than heroic single-lane speeds. The optimal value is therefore not a fixed number but a moving balance point, and historically, once the electronic-domain penalty steepens, parallelism wins.

## Notes

Jiamin Wu is an associate professor at the Department of Automation, Tsinghua University; Jingxuan Chen is an assistant professor at the Institute of Semiconductors, Chinese Academy of Sciences.

Jiamin Wu is an associate professor at the Department of Automation, Tsinghua University; Jingxuan Chen is an assistant professor at the Institute of Semiconductors, Chinese Academy of Sciences.

